# Effects of Digitalis on Generative Organs

**Published:** 1856-09

**Authors:** 


*Effects of Digitalis on Generative Organs.*—Mr. Brughmanns says, that if from 35 to 50 centigrammes of pulv. digitalis be given for five or six days, the most complete hyposthenizing effect is produced on the generative organs. He has thus given it with very great advantage to combat erotic excitement, whether due to excitable temperament, sedentary life, stimulant regimen, or the privation or excess of venereal pleasure, etc. He also finds it very useful in subduing the inflammatory accidents that so often accompany syphilitic diseases, and which may be prevented by its early admistration. It is pre-eminently useful when phymosis or paraphymosis, chordee, epididymitis, or adenitis are either present or feared.—*Med. Times and Gaz., from Rev. Méd. Chir.*

---

Paris, July 22d, 1856.

The striking differences which seem to exist between the genital organs of males and those of females in the superior classes of animals, and the differences which seem also to exist between the products of the secretion of the ovaries and that of the testicles, are merely appearances, and not true differences, according to two papers which have just been published here. In one of them, Professor Serres tries to prove that the series of metamorphoses which takes place in the seminal fluid, takes place also in the product of secretion of the ovaries. In the mother-cells of the semen, M. Serres finds the three elements of the primitive ovum, *i. e.*—1. An outside vesicle (the mother-cell of previous anatomists); 2. A small enclosed cell, corresponding with the germinal vesicle of the ovum; and, 3. A nucleus in this small cell, corresponding with the germinal spot of the ovum. The duplication of the enclosed cell in the semen takes place in the same manner as that of the germinal vesicle. In the semen, the cleaving of the cells is spontaneous; but, in the ovum, the cleaving of the germinal vesicle is not spontaneous, and takes place only after fecundation.

In a paper, communicated last week to the Société de Biologie, M. Charles Rouget, one of the most distinguished of the young French anatomists, has begun the exposition of researches upon the analysis between the male and the female genital organs. The object of this first paper is to show that the round ligament corresponds exactly with the gubernaculum testis. In point of structure, these two organs are alike; they are composed of blood-vessels and nerves coming from the same sources, and we find in them both striated muscular fibres coming from the same muscle. Besides these, there are many unstriped muscular fibres in the two organs. The analogy of these organs is very evident when we compare their mode of development in embryos. M. Rouget grounds his views on this subject upon researches made, not only on man, but on a great many species of mammals.

A communication, made by M. Cloez to the Académié des Sciences, shows that the re-agent employed to detect ozone in the atmosphere may lead to erroneous conclusions. This re-agent, iodole of potassium, may give the same reaction as with ozone, when it is exposed to the evaporation of nitric acid, and to the emanations from resinous trees and aromatic plants.

M. Briquet has read a paper on the elimination of quinine, at the same Academy. He has found that quinine passes very quickly into the urine. In half-an-hour after it has been ingested it is found in the urine; but the complete elimination of a dose requires many days. Urine seem to be the only secretion with which quinine is eliminated.

Dr. Brown-Séquard has shown to the Société de Biologie, that almost all the excretory ducts of glands, in birds, have rhythmical movements. Already Professor Claude Bernard had found that the choledochus and the pancreatic ducts have such movements.

Dr. Brown-Séquard has shown that the same thing exists in the vasa deferentia in adult birds. The movements in all these ducts are perfectly regular, and their rhythm seems to have no constant relation with that of the heart. He has ascertained that the number of contractions of each of these ducts in a given time, is not the same in one of them as in the others. The left vas deferens has not its contractions at the same time as the right. When the spinal cord is destroyed in all its length in a decapitated bird, the rhythmical movements continue for some time in the choledochus, pancreatic, and urinary ducts, and in the vasa deferentia, as well as in the heart. If the heart is taken away, all these excretory ducts still have, for a few minutes, strong rhythmical contractions. Dr. Brown-Séquard points out also the existence of rhythmical contractions of the trachea and bronchiæ in large sea-birds at each expiration. These contractions, which diminish the calibre of these tubes, contribute to the expulsion of the air.—*Paris Correspondence of the London Medical Times and Gazette.*

